# Axial Force Identification of Short Beam Members with Unknown Boundary Conditions Incorporating Rotational Inertia

**DOI:** 10.3390/s26134246

**Published:** 2026-07-04

**Authors:** Litian Liang, Bingjie Zhao, Yadong Yao, Jiammei Chang, Xin Guo

**Affiliations:** 1 School of Civil Engineering, Inner Mongolia University, Hohhot 010070, China; 2 Inner Mongolia Engineering Research Center of Testing and Strengthening for Bridges, Inner Mongolia University, Hohhot 010070, China

**Keywords:** beam theory, reissner energy equation, free vibration, modal analysis, axial force estimation

## Abstract

**Highlights:**

**What are the main findings?**

- Rotational inertia has a negligible influence on slender beam members but significantly affects the dynamic characteristics of short beams.
- The proposed method achieves reliable axial force identification under unknown boundary conditions through measured modal information.

**What are the implications of the main findings?**

- The developed formulation provides a more accurate theoretical framework for short beam structures where rotational inertia effects cannot be neglected.
- The proposed approach has potential applications in structural health monitoring and force evaluation of engineering beam structures with uncertain boundary conditions.

**Abstract:**

Accurate identification of axial forces in beam structures with unknown boundary conditions is important for structural assessment and safety monitoring. Most existing methods are based on Euler–Bernoulli beam theory and neglect the effect of rotational inertia. This simplification may reduce the accuracy of axial force identification for short beam members. To address this limitation, this study develops an axial force identification method that accounts for rotational inertia effects. First, a free-vibration governing equation for axially loaded beam members is derived based on the Reissner energy approach. Compared with the Euler–Bernoulli beam, the derived equation further accounts for the effect of rotational inertia. Then, based on the proposed dynamic formulation, an axial force identification method applicable to beam members with unknown boundary conditions is established by utilizing measured natural frequencies and mode shapes. Finally, the effectiveness and accuracy of the proposed method are systematically validated through both numerical simulations and experimental investigations on beam members. Numerical results indicate that incorporating rotational inertia improves axial force identification accuracy compared with conventional approaches, particularly for short beam members and higher-order modes. Experimental results further confirm its effectiveness, with a maximum identification error reduction of 7.69%.

## 1. Introduction

Axially loaded beam members are extensively employed in large-scale engineering structures owing to their high load-carrying efficiency and well-defined force-transfer mechanisms, as exemplified by space grid structures [1], offshore wind substations [2], and cable-stayed bridges [3]. As primary load-bearing components, their mechanical performance plays a decisive role in the overall safety and stability of the structure. Nevertheless, factors such as environmental corrosion, material degradation, and sustained loading may induce axial force deterioration or even structural failure. Therefore, the accurate identification of axial forces in such members is of critical importance for ensuring structural safety [4,5,6].

At present, vibration-based methods for axial force identification are widely adopted in engineering practice due to their non-destructive nature, cost-effectiveness, and rapid implementation [7,8,9,10]. The fundamental principle of these methods is that axial force or stress states can modify the dynamic characteristics of structures [11]. The resulting variations in modal parameters, such as natural frequencies and mode shapes, can be extracted from dynamic measurements and further utilized for structural assessment [12]. Early studies primarily relied on classical string theory to identify axial forces in slender members, benefiting from its simple formulation and relatively high accuracy [13]. This approach generally assumes ideal pinned boundary conditions and establishes an explicit relationship between natural frequencies and axial force. For example, Liu et al. [14] proposed a time-varying cable-force identification method. Instantaneous frequencies were extracted from cable vibration signals using adaptive chirp mode decomposition, and the cable force was estimated based on taut string theory.

However, the string theory neglects the effects of bending stiffness. As the slenderness ratio of the member decreases, the influence of bending stiffness on the structural dynamic behavior becomes more significant. Consequently, the string assumption may not be suitable for accurate axial force identification. To address this limitation, Euler–Bernoulli beam theory has been widely adopted for tension force identification, as it explicitly accounts for bending stiffness with satisfactory accuracy [15]. Bao et al. [16] identified time-varying cable tension forces by estimating the instantaneous modal frequencies of cable vibrations, while Zhang et al. [17] developed the STRICT algorithm for real-time cable tension identification based on instantaneous frequencies extracted from vibration responses. In addition, Guo et al. [18] proposed a target-free vision method to identify cable forces from modal frequencies extracted from cable vibrations. Nevertheless, such methods are typically derived on the basis of a certain boundary condition (fixed-fixed, hinged-hinged, or hinged-fixed) [19]. In practical engineering applications, however, the boundary conditions of structural members are often uncertain and generally lie between ideal clamped and pinned conditions. For beam members with large slenderness ratios, the influence of boundary conditions on axial force identification is often negligible. Therefore, frequency-based methods can still provide satisfactory accuracy. In contrast, for members with small slenderness ratios, boundary conditions significantly affect modal characteristics and therefore cannot be neglected. To address the issue of unknown boundary conditions, two main categories of methods have been developed. The first approach identifies axial forces by utilizing both natural frequencies and mode shapes of the beam member [20,21,22,23,24]. For instance, Rebecchi et al. [21] proposed an axial force identification method for slender beams with unknown boundary conditions by employing the Euler–Bernoulli beam model together with one natural frequency and the corresponding mode shape amplitudes measured at multiple locations. Similarly, Li et al. [22] used measurements from multiple sensors to simultaneously identify the axial force and the boundary stiffness at both ends, and was validated by numerical and experimental studies. Another approach introduces the concept of effective vibration length (EVL) to enable axial force identification under unknown boundary conditions [25,26,27,28,29,30,31]. For example, Chen et al. [25] proposed an explicit formulation analogous to that for hinged–hinged beams to estimate cable tension under unknown boundary conditions. In this approach, an EVL is determined for each vibration mode by fitting a sinusoidal function to mode shape ratios obtained from synchronized measurements at multiple locations. However, the accuracy of EVL-based methods deteriorates when measurements are concentrated near the cable–deck anchorage. To address this limitation, Chen et al. [29] further refined the approach for practical scenarios in which sensors are installed only near the lower cable end, thereby improving identification accuracy.

Most existing axial force identification methods for beam members with unknown boundary conditions are developed based on the Euler-Bernoulli beam theory. A representative example is the method proposed by Li et al. [22], in which the governing equation is derived from force equilibrium and the rotational inertia effect is neglected. This assumption is generally acceptable for beam members with large slenderness ratios, where the influence of rotational inertia on dynamic characteristics is negligible. However, as the slenderness ratio decreases, rotational inertia becomes increasingly significant and may reduce the accuracy of axial force identification. Therefore, these methods may not be suitable for short beam members with unknown boundary conditions. To overcome this limitation, a free-vibration governing equation for axially loaded beam members incorporating rotational inertia is derived in this study based on the Reissner energy approach [32]. The derived formulation is then applied to axial force identification of beam members with unknown boundary conditions.

The remainder of this paper is organized as follows. Section 2 presents the free-vibration governing equation of the axially loaded beam considering rotational inertia. Section 3 introduces the proposed axial force identification method for short beam members with unknown boundary conditions, explicitly accounting for rotational inertia effects. Section 4 provides numerical simulations to evaluate the accuracy and effectiveness of the proposed method. Section 5 further validates the approach through experimental studies on two beam members with relatively large and small slenderness ratios, respectively.

## 2. Improved Free Vibration Equation of Euler–Bernoulli Beam Under Axial Force

In this section, the displacement and stress condensation assumptions of the Euler–Bernoulli beam are introduced to establish the Reissner’s functional for a bending beam subjected to axial force. Based on extremum of functional, an improved governing equation for the free vibration of the member under axial force is derived. The fundamental theory of this method can be found in Refs. [23,24], and the detailed derivation is presented as follows.

### 2.1. Condensation Assumptions of Euler-Bernoulli Beam Displacement and Stress

**Figure 1:**
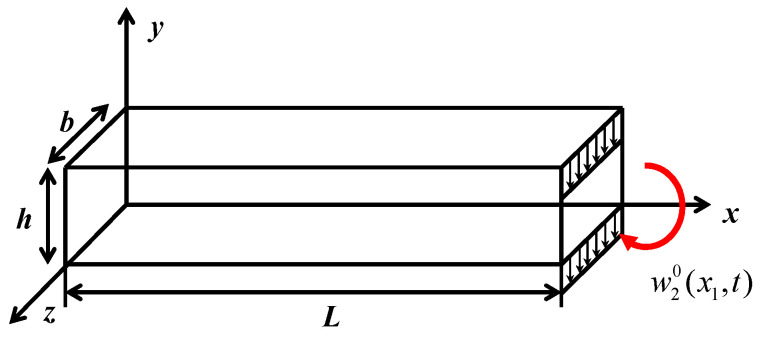
Transverse displacement $w_{2}^{0}(x,t)$.

**Figure 2:**
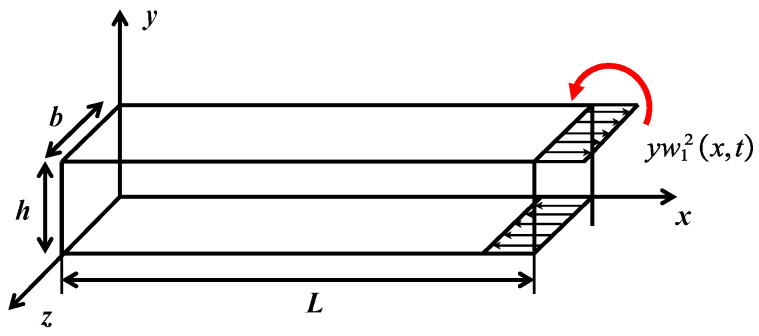
Longitudinal displacement $yw_{1}^{2}(x,t)$.

As illustrated in Figure 1 and Figure 2, when the beam undergoes bending deformation, longitudinal and transverse displacements are induced along the *x*- and *y*-directions, respectively. Under these kinematic conditions, the displacement condensation assumptions for the bending beam can be expressed as follows:

(1) $$\begin{array}{l} u(x,y,z,t)=yw_{1}^{2}(x,t)\\ w(x,y,z,t)=w_{2}^{0}(x,t)=w_{2}^{0} \end{array}$$

where $w_{1}^{2}(x,t)$ is the rotation angle of the beam cross-section.

Since the longitudinal and transverse displacements generate axial and shear stresses, respectively, and the Euler–Bernoulli beam theory neglects shear deformation, only the axial stress is retained in the planar beam model. Accordingly, the stress condensation can be written as follows:

(2) $$\begin{array}{l} \sigma_{11}(x,y,z,t)=y\sigma_{11}^{2}(x,t)=y\sigma_{11}^{2}\\ \sigma_{12}(x,y,z,t)=\sigma_{12}^{0}(x,t)=0 \end{array}$$

In addition, under the plane-section assumption, the transverse displacement of the beam is related to the cross-sectional rotation as follows:

(3) $$w_{1}^{2}(x,t)=-\frac{\partial w_{2}^{0}(x,t)}{\partial x}$$

From Equations (1)–(3), it follows that the displacement field of the planar beam involves only one independent variable $w_{2}^{0}$, and the stress field is also governed by a single unknown $\sigma_{11}^{2}$. Therefore, the free vibration problem of the bending beam is reduced to solving for two unknown variables.

### 2.2. Reissner’s Functional for a Bending Beam Under Axial Force

To establish the relationship between transverse displacement, longitudinal stress, and axial force, Reissner’s functional is employed to derive the governing equations for the free vibration of a bending beam. The general form of the Reissner’s functional for a beam is defined as follows

(4) $$R(w_{i},\sigma_{ij})=\int_{t}\int_{V}(\frac{1}{2}p{\left(\frac{\partial w_{i}}{\partial t}\right)}^{2}-\sigma_{ij}\varepsilon_{ij}+\frac{1}{2}\sigma_{ij}S_{ijkl}\sigma_{kl}+f_{i}w_{i})dVdt$$

where $R(w_{i},\sigma_{ij})$ denotes the total energy of the beam, *w_i_* represents the displacement components, *σ_ij_* and *ε*_ij_ are the stress and strain components, respectively, *p* is the mass density of the beam, *f_i_* denotes the body force components, which are neglected under free vibration conditions, *S_ijkl_* is the flexibility coefficient, and *V* is the volume of the beam.

Within the Euler–Bernoulli beam framework, the displacement and stress fields are defined according to the condensation assumptions. Substituting these fields into the Reissner functional yields the following energy expression:

(5) $$R(w_{2}^{0},\sigma_{11}^{2})=\frac{1}{2}\int_{t}\int_{0}^{L}\left[pl{\left(\frac{\partial^{2}w_{2}^{0}}{\partial x\partial t}\right)}^{2}+pA{\left(\frac{\partial w_{2}^{0}}{\partial t}\right)}^{2}-I\sigma_{11}^{2}\frac{\partial^{2}w_{2}^{0}}{\partial x^{2}}+IS_{1111}{\left(\sigma_{11}^{2}\right)}^{2}\right]dxdt$$

where *L* is the beam length and *A* is the cross-sectional area.

It should be noted that the Reissner functional given in Equation (5) does not account for the effect of axial tension. To incorporate this effect, the potential energy associated with the axial force *N* is introduced. For a bending beam, the corresponding energy contribution can be expressed as follows

(6) $$R_{N}=-\int_{0}^{L}N(\frac{\partial^{2}w_{2}^{0}}{\partial x^{2}}+\frac{1}{2}{\left(\frac{\partial w_{2}^{0}}{\partial x}\right)}^{2})dx$$

By combining Equations (4) and (5), the Reissner functional for the bending beam, expressed in terms of the displacement, axial stress, and axial tension *N*, can be formulated as follows:

(7) $$R(w_{2}^{0},\sigma_{11}^{2},N)=\frac{1}{2}\int_{t}\int_{0}^{L}[pl{\left(\frac{\partial^{2}w_{2}^{0}}{\partial x\partial t}\right)}^{2}+pA{\left(\frac{\partial w_{2}^{0}}{\partial t}\right)}^{2}-I\sigma_{11}^{2}\frac{\partial^{2}w_{2}^{0}}{\partial x^{2}}+IS_{1111}{\left(\sigma_{11}^{2}\right)}^{2}-N(\frac{\partial^{2}w_{2}^{0}}{\partial x}+\frac{1}{2}{\left(\frac{\partial w_{2}^{0}}{\partial x}\right)}^{2})]dxdt$$

### 2.3. Extremum Principle of the Reissner Functional for a Bending Beam

A general functional describing the vibration of a beam in terms of time and displacement can be expressed as

(8) $$L(w(x,t))=\int_{t}\int_{0}^{L}F(w(x,t))dxdt$$

Define $w_{,t}=\frac{\partial w}{\partial t},w_{,x}=\frac{\partial w}{\partial x},w_{,tx^{j}}=\frac{\partial^{j+1}w}{\partial t\partial x^{j}}$, where the index *j* ranges from 1 to *n*. Then, the extreme value of w(x,t) in the above functional can be written as follows

(9) $$\frac{\partial F}{\partial w}-\frac{\partial }{\partial t}\frac{\partial F}{\partial w_{,t}}-\frac{\partial }{\partial x}\frac{\partial F}{\partial w_{,x}}+\ldots +{\left(-1\right)}^{n}\frac{\partial^{n+1}}{\partial t\partial x^{n}}\frac{\partial F}{\partial w_{,tx}}+\frac{\partial F}{\partial w_{,tx^{n}}}=0$$

Equation (7) shows that the highest-order derivative in the energy functional is second order. Accordingly, Equation (9) can be rewritten as follows:

(10) $$\frac{\partial R}{\partial w}-\frac{\partial }{\partial t}\frac{\partial R}{\partial w_{,t}}-\frac{\partial }{\partial x}\frac{\partial R}{\partial w_{,x}}+\frac{\partial^{2}}{\partial t\partial x}\frac{\partial R}{\partial w_{,tx}}+\frac{\partial^{2}}{\partial x^{2}}\frac{\partial R}{\partial w_{,xx}}=0$$

### 2.4. Improved Governing Equation for Free Vibration of the Euler–Bernoulli Beam Under Axial Force

Based on the Reissner functional established above, the governing equations of the beam under axial tension are derived by enforcing the extremum principle. The stationary condition of the functional with respect to each independent variable yields the corresponding dynamic equations. Taking the variation with respect to the displacement variable $w_{2}^{0}$ yields

(11) $$-pA\frac{\partial^{2}w_{2}^{0}}{\partial t^{2}}+pI\frac{\partial^{4}w_{2}^{0}}{\partial x^{2}\partial t^{2}}-I\frac{\partial^{2}\sigma_{11}^{2}}{\partial x^{2}}+N\frac{\partial^{2}w_{2}^{0}}{\partial x^{2}}=0$$

Taking the variation with respect to the stress variable $\sigma_{11}^{2}$ yields

(12) $$-I\frac{\partial^{2}w_{2}^{0}}{\partial x^{2}}+IS_{1111}\sigma_{11}^{2}=0$$

From Equation (12), the relationship between $w_{2}^{0}$ and $\sigma_{11}^{2}$ can be written as

(13) $$\sigma_{11}^{2}=\frac{1}{S_{1111}}\frac{\partial^{2}w_{2}^{0}}{\partial x^{2}}$$

By substituting Equation (13) into Equation (11), the governing equation for the transverse displacement $w_{2}^{0}$ of the bending beam is obtained as follows:

(14) $$-pA\frac{\partial^{2}w_{2}^{0}}{\partial t^{2}}+pI\frac{\partial^{4}w_{2}^{0}}{\partial x^{2}\partial t^{2}}-\frac{1}{S_{1111}}I\frac{\partial^{4}w_{2}^{0}}{\partial x^{4}}+N\frac{\partial^{2}w_{2}^{0}}{\partial x^{2}}=0$$

For simplicity, the following notations are adopted: *E* = 1/*S*_1111_, where *E* is the Young’s modulus and $w_{2}^{0}=w$. The governing equation for the free vibration of the beam under axial tension is given by

(15) $$EI\frac{\partial^{4}w}{\partial x^{4}}+pA\frac{\partial^{2}w}{\partial t^{2}}-N\frac{\partial^{2}w}{\partial x^{2}}-pI\frac{\partial^{4}w}{\partial x^{2}\partial t^{2}}=0$$

It should be noted that the rotational inertia term in Equation (15) originates from the corresponding rotational inertia component included in the Reissner energy functional in Equation (5). As indicated by Equation (15), the proposed governing equation extends the classical free vibration model of a tensioned member by incorporating the effect of rotational inertia $pI\frac{\partial^{4}w}{\partial x^{2}\partial t^{2}}$.

## 3. Axial Force Identification in Beams with Unknown Boundary Conditions Based on the Proposed Governing Equation

This section addresses the problem of axial force identification in beams with unknown boundary conditions based on the proposed governing equation. The formulation follows Ref. [22] and is extended to account for the effect of rotational inertia.

By applying the method of separation of variables, the solution to Equation (15) is assumed in the form

(16) w(x,t)=ϕ(x)q(t)

where ϕ(x) denotes the mode shape of the beam, and *q*(*t*) is the generalized coordinate representing the time-dependent amplitude. Substituting Equation (16) into Equation (15) leads to the following expression

(17) $$EI\phi^{⁗}(x)q(t)+pA\phi (x)\overset{..}{q}(t)-N\phi^{\prime \prime }(x)q(t)-pI\phi^{\prime \prime }(x)\overset{..}{q}(t)=0$$

where “ . ”and “ ’ ”denote derivatives with respect to time *t* and spatial coordinate *x*, respectively. Higher-order derivatives are defined in an analogous manner.

By simplifying Equation (17), the following expression is obtained

(18) $$\frac{EI\phi^{⁗}(x)-N\phi^{\prime \prime }(x)}{pA\phi (x)-pI\phi^{\prime \prime }(x)}=-\frac{\ddot{q}(t)}{q(t)}$$

Equation (17) can be satisfied for all *t* and *x* only if both sides are equal to a constant. Letting this constant frequency be $\omega^{2}$, Equation (17) reduces to two independent ordinary differential equations

(19) $$\ddot{q}(t)+w^{2}q(t)=0$$

(20) $$EI\phi^{⁗}(x)+(pIw^{2}-N)\phi^{\prime \prime }(x)-pA\phi (x)=0$$

Simplifying Equation (20) yields

(21) $$\phi^{⁗}(x)+g\phi^{\prime \prime }(x)-a^{4}\phi (x)=0$$

where the symbols *g* and *a*^4^ are defined as

(22) $$g=\frac{pIw^{2}-N}{EI},a^{4}=\frac{pAw^{2}}{EI}$$

Assuming a solution of the form $\phi (x)=Ce^{sx}$, substitution into Equation (21) yields the characteristic equation

(23) $$(s^{4}+gs^{2}-a^{4})Ce^{sx}=0$$

Solving the characteristic equation yields the roots s, which can be expressed as

(24) $$s_{1,2}=\pm i\delta ;s_{3,4}=\pm \varepsilon$$

where

(25) $$\delta =\sqrt{{\left(a^{4}+\frac{g^{2}}{4}\right)}^{\frac{1}{2}}+\frac{g}{2}};\varepsilon =\sqrt{{\left(a^{4}+\frac{g^{2}}{4}\right)}^{\frac{1}{2}}-\frac{g}{2}}$$

Substituting the roots into the general solution and recasting the exponential terms into trigonometric and hyperbolic forms, the general solution is obtained as

(26) $$\phi (x)=C_{1}\sin\delta x+C_{2}\cos\delta x+C_{3}\sinh\varepsilon x+C_{4}\cosh\varepsilon x$$

where the coefficients *C*_1_–*C*_4_ governed by the boundary conditions.

Given the structural properties and a measured natural frequency ω of the beam, Equation (26) involves five unknowns, including the axial force and the boundary coefficients. To eliminate the influence of boundary conditions on the axial force identification, the modal displacement ratio $\lambda_{ij}$ is introduced. By measuring the mode shape at five locations along the beam and selecting one as a reference point, four independent displacement ratios can be constructed as

(27) $$\lambda_{ij}=\frac{\phi (x_{i})}{\phi (x_{j})}=\frac{C_{1}\sin\delta x_{i}+C_{2}\cos\delta x_{i}+C_{3}\sinh\varepsilon x_{i}+C_{4}\cosh\varepsilon x_{i}}{C_{1}\sin\delta x_{j}+C_{2}\cos\delta x_{j}+C_{3}\sinh\varepsilon x_{j}+C_{4}\cosh\varepsilon x_{j}}\;(j=1,2,3,4)$$

where $\phi (x_{i})$ and $\phi (x_{j})$ denote the mode shapes values at the reference point and *i*-th point, respectively. Substituting the modal shape into the displacement ratios and rearranging, a system of linear homogeneous equations with respect to *C*_1_–*C*_4_ is obtained. Collecting these equations lead to the compact matrix form

(28) A(N)C=0

where $C={\left[C_{1}\;C_{2}\;C_{3}\;C_{4}\right]}^{T}$, **A**(*N*) is the coefficient matrix and its explicit form is given by

(29) $$A(N)=\left[\begin{array}{cccc} \lambda_{i1}\sin\delta x_{1}-\sin\delta x_{i} & \lambda_{i1}\cos\delta x_{1}-\cos\delta x_{i} & \lambda_{i1}\sinh\varepsilon x_{1}-\sinh\varepsilon x_{i} & \lambda_{i1}\cosh\varepsilon x_{1}-\cosh\varepsilon x_{i}\\ \lambda_{i2}\sin\delta x_{2}-\sin\delta x_{i} & \lambda_{i2}\cos\delta x_{2}-\cos\delta x_{i} & \lambda_{i2}\sinh\varepsilon x_{2}-\sinh\varepsilon x_{i} & \lambda_{i2}\cosh\varepsilon x_{2}-\cosh\varepsilon x_{i}\\ \lambda_{i3}\sin\delta x_{3}-\sin\delta x_{i} & \lambda_{i3}\cos\delta x_{3}-\cos\delta x_{i} & \lambda_{i3}\sinh\varepsilon x_{3}-\sinh\varepsilon x_{i} & \lambda_{i3}\cosh\varepsilon x_{3}-\cosh\varepsilon x_{i}\\ \lambda_{i4}\sin\delta x_{4}-\sin\delta x_{i} & \lambda_{i4}\cos\delta x_{4}-\cos\delta x_{i} & \lambda_{i4}\sinh\varepsilon x_{4}-\sinh\varepsilon x_{i} & \lambda_{i4}\cosh\varepsilon x_{4}-\cosh\varepsilon x_{i} \end{array}\right]\;$$

It is noted that **A**(*N*) depends on the axial force through the modal parameters of the beam. For the existence of nontrivial solutions, the determinant of the coefficient matrix must vanish, which yields a nonlinear equation with respect to the axial force *N*.

(30) $$\left|A(N)\right|=0$$

Therefore, the axial force *N* can be identified by solving the above equation. Accordingly, the proposed method enables axial force identification without prior knowledge of boundary conditions while accounting for the effect of rotational inertia.

## 4. Numerical Verification

**Figure 3:**
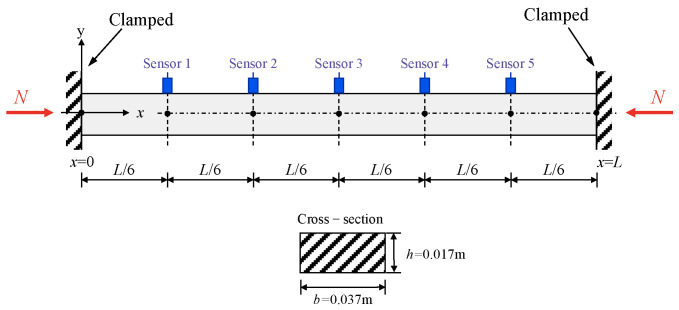
The test bar model.

To validate the proposed governing equation and assess the applicability of the axial force identification method, a finite element model of the beam is established using ANSYS 2022 (ANSYS, Inc., Canonsburg, PA, USA), as shown in Figure 3. The bar is subjected to clamped–clamped boundary conditions, with the axial tensile force *N* applied at both ends. The material and geometric properties of the beam are specified as follows: Young’s modulus *E* = 210 GPa, density *p* = 7860 kg/m^3^, and Poisson’s ratio *v* = 0.3. The beam has a rectangular cross-section with width *b* = 0.037 m and height h = 0.017 m. Suppose that five sensors are uniformly distributed along the bar. The modal characteristics, including natural frequencies and corresponding mode shapes, are obtained from the finite element model.

The influence of rotational inertia on axial force identification is investigated through numerical validations. Different vibration modes, axial force levels, and member lengths are considered. The results are compared with those obtained from the formulation that neglect rotational inertia.

### 4.1. Different Modes

First, a beam with a length of 0.66 m is considered, subjected to an axial tensile force of 20 kN. Modal analysis is performed to obtain the first three natural frequencies and the corresponding mode shapes of the beam. The axial force identification results for different modes are summarized in Table 1.

As shown in Table 1, the conventional formulation in [22] yields increasing identification errors with modal order, from 19.208 kN to 17.347 kN, whereas the proposed formulation accounting for rotational inertia reduces the corresponding results to 19.421 kN and 18.902 kN, respectively. These results demonstrate that consideration of rotational inertia improves the identification accuracy. Moreover, the improvement becomes more pronounced at higher modes, indicating that the influence of rotational inertia increases with modal order.

### 4.2. Different Axial Force

This section investigates the influence of axial force levels on the identification performance of the proposed formulation accounting for rotational inertia. The same beam as described above is considered, and the axial tensile force is varied from 15 kN to 25 kN with an increment of 5 kN. The first-mode natural frequencies and corresponding mode shapes at the sensor locations are extracted under different axial force conditions, and the corresponding identification results are listed in Table 2.

As shown in Table 2, the conventional formulation exhibits decreasing identification errors with increasing axial force, with the maximum error of −5.33%. In contrast, the proposed formulation consistently provides improved accuracy across all conditions. The identified axial forces are 14.410 kN, 19.421 kN, and 25.479 kN for axial forces of 15, 20, and 25 kN, respectively, with a maximum error of −3.93%. The improvement is more evident at low axial force levels. This is because the rotational inertia term has a larger relative contribution to the dynamic response when the axial force level is low. Therefore, neglecting rotational inertia may introduce larger errors in axial force identification.

### 4.3. Different Length

To investigate the influence of the slenderness ratio on axial force identification accuracy, a series of beam members with identical cross-sectional dimensions but different lengths are considered. The beam length is varied from 0.66 m to 0.84 m, while a constant axial tensile force of 15 kN is applied to all cases. The axial force identification is performed using the modal data associated with the first bending mode of the beam members, and the identified axial forces for different beam lengths are summarized in Table 3.

For the short beam member with a length of 0.66 m, the method in [22] yields an identified axial force of 14.200 kN, corresponding to a relative error of 5.33%, whereas the proposed method reduces the error to 3.93% with the identified axial force of 14.410 kN. As the beam length increases from 0.66 m to 0.84 m, the identification errors of two methods gradually decrease. For the beam member with a length of 0.84 m, the relative errors of two methods are only 1.43% and 1.99%, respectively. The error reduction decreases from 1.40% to 0.56% as the beam length increases from 0.66 m to 0.84 m. These results indicate that rotational inertia significantly affects the axial force identification accuracy of short beam structures with relatively small slenderness ratios, whereas its influence gradually diminishes as the slenderness ratio increases. This phenomenon can be attributed to the increasing influence of rotational inertia. The contribution of the rotational inertia term is proportional to the square of the natural frequency, and short beam members generally have higher natural frequencies. Therefore, incorporating rotational inertia can effectively improve the identification accuracy for short beam members, thereby demonstrating the correctness and necessity of the proposed formulation.

## 5. Experimental Verification

In this section, two laboratory experiments are conducted to further validate the proposed algorithm. A slender rectangular bar is first considered, where the effect of rotational inertia is relatively small. A hollow rod is then examined, in which the influence of rotational inertia becomes more pronounced. This experimental design allows for a comprehensive assessment of the proposed method under varying levels of rotational inertia.

### 5.1. Test 1—Solid Rectangular Bar

The experimental data are obtained from [22]. The bar has a rectangular cross-section with width *b* = 0.035 m and height *h* = 0.005 m, and the length of *L* = 0.72 m. The material is steel, with density and Young’s modulus taken as 7800 kg/m^3^ and 210 GPa, respectively. Five A353B66 accelerometers (sensitivity 0.1 V/g, mass 1.5 g) are uniformly distributed along the beam with a spacing of 0.12 m. The structure is excited by a single-point hammer impact, and the measured acceleration responses are processed using the stochastic subspace identification method to extract the first five natural frequencies and the corresponding normalized modal displacement ratios at the sensor locations. Detailed experimental data can be found in [22]. Table 4 presents the axial force identification results and corresponding relative errors for the first and third modes under different loading conditions, respectively.

As shown in Table 4, for the first mode, the identification results obtained using the proposed formulation are very close to those based on the classical Euler–Bernoulli beam theory, with the marginal improvement in accuracy of less than 0.01%. For the third mode, the improvement remains limited. For instance, when the actual axial force is 10 kN, the identified value is 9.593 kN, corresponding to an error of −4.07%, with an accuracy improvement of only 0.12%. The improvement in identification accuracy is limited for the present test case. This is attributed to the small influence of rotational inertia in the tested slender beam, which is consistent with the numerical results obtained previously. Nevertheless, the experimental results still demonstrate the validity of the proposed formulation and its applicability to axial force identification.

### 5.2. Test 2—A Hollow Rod

**Figure 4:**
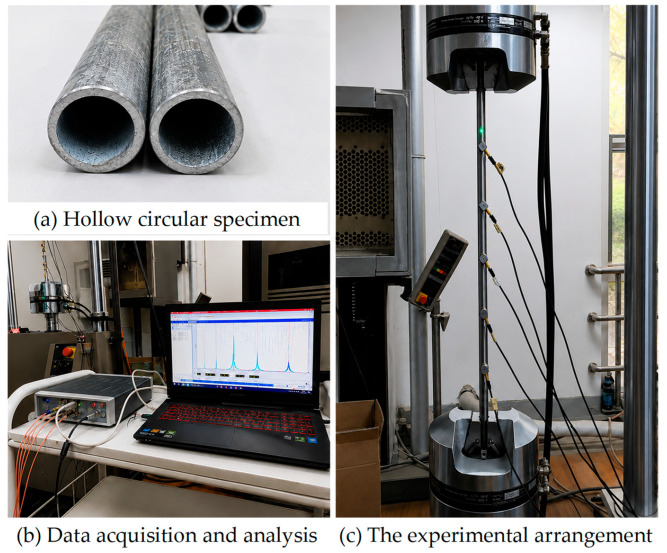
Photograph of the experiment [33].

To further investigate the performance of the proposed method under conditions where rotational inertia plays a significant role, a hollow circular beam specimen with a relatively small slenderness ratio is considered. The experimental data were obtained from previous work by the author research group [33], as shown in Figure 4, The specimen is made of 304 stainless steel, with density *p* = 7930 kg/m^3^ and Young’s modulus *E* = 193 GPa. The rod has a length of 0.6 m, an outer diameter of 0.019 m, and a wall thickness of 0.004 m. Five accelerometers (sensitivity 100 mV/g, mass 1.5 g) are uniformly distributed along the rod. Although both ends of the specimen are designed as fixed supports, the actual boundary condition inevitably deviates from an ideal fixed constraint and can be regarded as being between fixed and pinned supports. The axial force is varied from 10 to 30 kN, and at each load level, the bar is excited by a hammer impact to extract the modal parameters, including natural frequencies and mode shapes. The estimated axial forces obtained from the first two modes for all loading cases are summarized in Table 5.

As shown in Table 5, the proposed formulation provides comparable results to the reference method. For the first mode, the improvement is relatively small, with a maximum improvement of 1.37%. This can be attributed to the limited contribution of rotational inertia in the first mode. In contrast, for the second mode, the proposed method achieves a clearer reduction in identification errors for the second mode, with improvements ranging from approximately 2.83% to 7.69%. This difference is mainly related to the increased influence of rotational inertia in higher-order modes. The experimental results show similar trends to the numerical results, and the influence of rotational inertia is more evident for higher-order modes and low axial force levels. These results indicate that the proposed formulation is more suitable for short beam members with low slenderness ratios, where the rotational inertia effect has a greater influence on axial force identification. These results indicate that the proposed formulation provides greater advantages for short beam members.

### 5.3. Discussion

The numerical and experimental results indicate that the influence of rotational inertia on axial force identification is not always negligible. For the lower-order modes of slender beam members, the influence of rotational inertia is very small, and the conventional identification method can still provide satisfactory accuracy. However, the influence of rotational inertia becomes more significant as the modal order increases. It is also more pronounced for short beam members. Neglecting this effect under these conditions may lead to noticeable errors in axial force identification. Therefore, rotational inertia should be considered to accurately identify axial forces in short beam members

The proposed method extends the applicability of vibration-based axial force identification to short beam members with unknown boundary conditions. It accounts for the effect of rotational inertia without requiring prior knowledge of boundary conditions. As a result, higher identification accuracy can be achieved for short beam members.

## 6. Conclusions

This study establishes a beam dynamic model under axial force that accounts for the effect of rotational inertia and further proposes an axial force identification method for beam structures with unknown boundary conditions. Numerical simulations and experimental investigations are conducted to validate the effectiveness and accuracy of the proposed method. The main conclusions are summarized as follows:

(1) An improved free-vibration equation for the beam under axial force was developed based on the Euler beam theory and the Reissner’s energy formulation, in which the effect of rotary inertia was taken into account.
(2) Based on the developed governing equation, an axial force identification method accounting for rotational inertia was proposed for beam members with unknown boundary conditions.
(3) The accuracy and applicability of the proposed method were validated through numerical simulations and experimental investigations. The results show that the influence of rotational inertia is negligible for slender beam members. However, for short beam members, the rotational inertia effect becomes significant and should be considered in axial force identification.

The present study also has some limitations. This study focuses on the influence of rotational inertia on axial force identification. Therefore, measurement noise and modal identification uncertainty were not considered in the present analysis. Future work will develop vision-based axial force identification methods. The effects of measurement noise and modal identification uncertainty on the identification results will be investigated.

## Figures and Tables

**Table 1:** The estimated axial forces of the bar for different modes.

	Identification Results	Modal Order		
		1st	2nd	3rd
This paper	Axial force(kN)	19.421	19.254	18.902
	Error (%)	−2.90	−3.73	−5.49
Reference [22]	Axial force (kN)	19.208	18.323	17.347
	Error(%)	−3.96	−8.39	−13.27

**Table 2:** The estimated axial forces of the bar for different axial force levels.

	Identification Results	Axial Force (kN)		
		15	20	25
This paper	Axial force(kN)	14.410	19.421	24.479
	Error (%)	−3.93	−2.90	−2.08
[22]	Axial force (kN)	14.200	19.208	24.262
	Error (%)	−5.33	−3.96	−2.95

**Table 3:** The estimated axial forces of the bar for different length.

	Identification Results	Length (m)			
		0.66	0.72	0.78	0.84
This paper	Axial force (kN)	14.410	14.636	14.695	14.785
	Error (%)	−3.93	−2.43	−2.03	−1.43
[22]	Axial force (kN)	14.200	14.486	14.585	14.702
	Error (%)	−5.33	−3.43	−2.77	−1.99
	Error reduction (%)	1.40	1.00	0.74	0.56

**Table 4:** The axial force identification results for the first and third modes under different axial force levels.

Modal Order	Cases	Identification Results				
		This Paper (kN)	Error (%)	[22] (kN)	Error (%)	Improved Accuracy (%)
1	10	9.711	−2.89	9.711	−2.89	0.00
	15	14.501	−3.32	14.501	−3.33	0.01
	20	19.330	−3.35	19.329	−3.36	0.01
	25	24.331	−2.68	24.329	−2.68	0.00
3	10	9.593	−4.07	9.581	−4.19	0.12
	15	14.374	−4.17	14.361	−4.26	0.09
	20	19.354	−3.23	19.338	−3.31	0.08
	25	24.171	−3.32	24.153	−3.39	0.07

**Table 5:** The axial force identification results for the first two modes under different axial force levels.

Modal Order	Cases	Identification Results				
		This Paper (kN)	Error (%)	[22] (kN)	Error (%)	Improved Accuracy (%)
1	10	10.037	0.37	9.93	−0.70	−0.33
	15	15.094	0.63	14.983	−0.11	0.51
	20	19.93	−0.35	19.814	−0.93	0.58
	25	24.994	−0.02	24.874	−0.50	0.48
	30	29.933	−0.22	29.521	−1.60	1.37
2	10	9.503	−4.97	8.734	−12.66	7.69
	15	14.495	−3.37	13.706	−8.63	5.26
	20	19.519	−2.41	18.710	−6.45	4.04
	25	23.813	−4.75	22.521	−9.92	5.17
	30	29.521	−1.60	28.673	−4.42	2.83

## Data Availability

The original contributions presented in this study are included in the article. Further inquiries can be directed to the corresponding author.
